# Bibliometric and visualized analysis of the relationship between rheumatoid arthritis and periodontitis-related bacteria using CiteSpace software

**DOI:** 10.3389/fmicb.2025.1589331

**Published:** 2025-07-16

**Authors:** Tianqiong Lin, Zehong Xu, Jiali Chen, Xiaoyu Wang, Qiaoping Li, Biying Ye, Chaoyan Hu

**Affiliations:** ^1^Department of Nursing, The Third Affiliated Hospital of Guangzhou University of Chinese Medicine, Guangzhou, China; ^2^Department of Rheumatology, The Third Affiliated Hospital of Guangzhou University of Chinese Medicine, Guangzhou, China; ^3^Department of Orthopedics, The Third Affiliated Hospital of Guangzhou University of Chinese Medicine, Guangzhou, China; ^4^Department of General Surgery, The First Affiliated Hospital of Guangdong Pharmaceutical University, Guangzhou, China

**Keywords:** bibliometrics, rheumatoid arthritis, oral microbiome, inflammation, *Porphyromonas gingivalis*

## Abstract

**Background:**

Rheumatoid arthritis (RA) is a chronic systemic autoimmune disease of unknown etiology. Recent studies have indicated a potential relationship between the oral microbiome and the onset and progression of RA. However, research trends in this area have not been comprehensively examined. The aim of this study was to conduct a bibliometric analysis of the relationship between RA and the oral microbiome from January 1, 1995, to January 10, 2024, to elucidate the research landscape, including hot topics and emerging trends.

**Methods:**

We extracted literature related to RA and the oral microbiome from the Web of Science database. Utilizing CiteSpace software, we analyzed publications, countries, institutions, authors, and keywords through a visual knowledge graph to assess research hotspots and trends.

**Results:**

In total, 833 articles were identified, revealing a consistent increase in the number of annual publications in this field over the study period. The United States has emerged as the leading country in terms of publication volume, with Harvard University being the most prolific institution. Among the authors, Jan Potempa has the highest number of publications. Keyword analysis indicated that current research hotspots concerning the relationship between RA and the oral microbiome primarily focus on *Porphyromonas gingivalis*, periodontitis, inflammation, expression, and peptidylarginine deiminase. Investigating the mechanisms by which oral and intestinal microorganisms influence RA, as well as developing intervention strategies targeting these microbiotas, is anticipated to be a significant future research direction.

**Conclusion:**

This study characterized the trends in the literature regarding the relationship between RA and the oral microbiome, providing valuable insights for scholars pursuing further research.

## 1 Introduction

Rheumatoid arthritis (RA) is a systemic autoimmune disease with a global prevalence of approximately 0.5 to 1%. It is characterized by chronic inflammatory destruction of joint tissue, which arises from an abnormal autoimmune response influenced by genetic predisposition, environmental factors, and the interplay between epigenetics and post-translational modifications. The primary symptoms of RA include morning stiffness, joint pain, and swollen joints. As the disease progresses, the joint damage becomes gradual and irreversible, potentially leading to disability (Kobayashi and Bartold, 2023). According to the projected demographic changes, researchers estimate that by 2050, there will be 31.7 million individuals with RA worldwide, representing an 80.2% increase in cases from 2020 to 2050 (GBD 2021 Rheumatoid Arthritis Collaborators, 2023).

However, the pathogenesis of RA remains unclear. However, as research progresses, bacteria are increasingly being recognized as potentially related to RA. In particular, the microbiota of the alimentary tract, in conjunction with host factors associated with RA and various environmental influences, presents unexplored potential effector mechanisms for the development of RA (Wells et al., 2019). The oral cavity, as the initial segment of the alimentary tract, hosts billions of bacteria comprising over 700 identified species, some of which may trigger autoimmune responses associated with RA (Peng et al., 2022). Periodontitis (PD), an inflammatory disease caused by oral bacteria, is associated with RA. Notably, *Porphyromonas gingivalis* (*P. gingivalis*), an oral bacterium associated with PD, is associated with RA. *P. gingivalis* and gingival tissues affected by PD are thought to initiate citrulline-specific autoimmune responses, which are characterized by antibody reactions to citrullinated proteins (Kobayashi and Bartold, 2023). In addition to *P. gingivalis*, other oral bacteria such as *Actinomycetes* (Konig et al., 2016), *Prevotella intermedia* (Schwenzer et al., 2017), *Fusobacterium nucleatum* (Eriksson et al., 2022), *Tannerella forsythia* (Manoil et al., 2021), and *Megasphaera* spp. (Manoil et al., 2021), have also been implicated in RA. Research has indicated that certain components of the oral microbiome may influence immune responses through their virulence mechanisms, contributing to the onset and progression of RA (GBD 2021 Rheumatoid Arthritis Collaborators, 2023). Therefore, gaining insights into the current research hotspots and emerging trends in the relationship between RA and the oral microbiome is critical for advancing the field. Unlike previous reviews that primarily focus on the impact of RA on oral health, this study employs a dual approach by analyzing both oral and gut microbiota in the context of RA pathogenesis. This broader perspective allows us to explore the interconnected roles of microbial communities in systemic diseases, thereby addressing gaps in existing literature and paving the way for innovative therapeutic strategies.

Bibliometrics is a discipline that applies quantitative approaches, such as mathematics and statistics, to systematically analyze the distribution patterns, quantitative relationships, and internal connections among documents, based on their measurable characteristics (Cooper, 2015). CiteSpace software was utilized to generate and analyze co-citation networks from bibliographic records retrieved from the Web of Science (Zhou et al., 2023). This tool not only visualizes research hotspots and knowledge structures but also enables the identification of temporal trends, keyword clusters, and collaborative networks, which are critical for understanding the evolving landscape of RA and oral microbiome research. Unlike traditional review methods, our approach leverages advanced bibliometric techniques to provide a more nuanced and systematic analysis of the field, distinguishing this study from prior reviews such as the one by Dagli et al. (2024). This tool effectively visualizes results, enabling researchers to identify research hotspots, map knowledge structures, explore collaborative relationships, and forecast future directions within a specific domain. It thereby offers a strong foundation for the formulation of clinical guidelines (Tian et al., 2017).

In this study, CiteSpace was employed to perform a bibliometric analysis of annual publications and to determine the most productive and influential countries, institutions, authors, and journals. Furthermore, a keyword analysis was conducted to investigate the current state, research hotspots, and emerging trends in the correlation between RA and the oral microbiome.

## 2 Materials and methods

### 2.1 Data extraction and preprocessing

Literature pertaining to RA and the oral microbiome was retrieved from the Web of Science,^1^ covering publications from January 01, 1995, to January 10, 2024. The search strategy was as follows.

#1: TS = Arthritis, rheumatoid OR rheumatoid arthritis. #2: TS = oral flora OR oral microbiome OR oral microbiota OR oral microbiomes OR oral bacteria OR *Porphyromonas gingivalis* OR *Bacteroides gingivalis* OR *P. gingivalis* OR *Porphyromonas* OR *Prevotella* OR *Prevotella intermedia* OR *P. intermedia* OR *Aggregatibacter actinomycetemcomitans* OR *Actinobacillus actinomycetemcomitans* OR *Actinomycetemcomitans* OR *Fusobacterium nucleatum* OR *Treponema denticola* OR *Tannerella forsythia* OR *Filifactor alocis* OR *Leptotrichia*. #1 AND #2. Original articles and review articles were selected as document types, and literature published in English was included.

**Figure 1:**
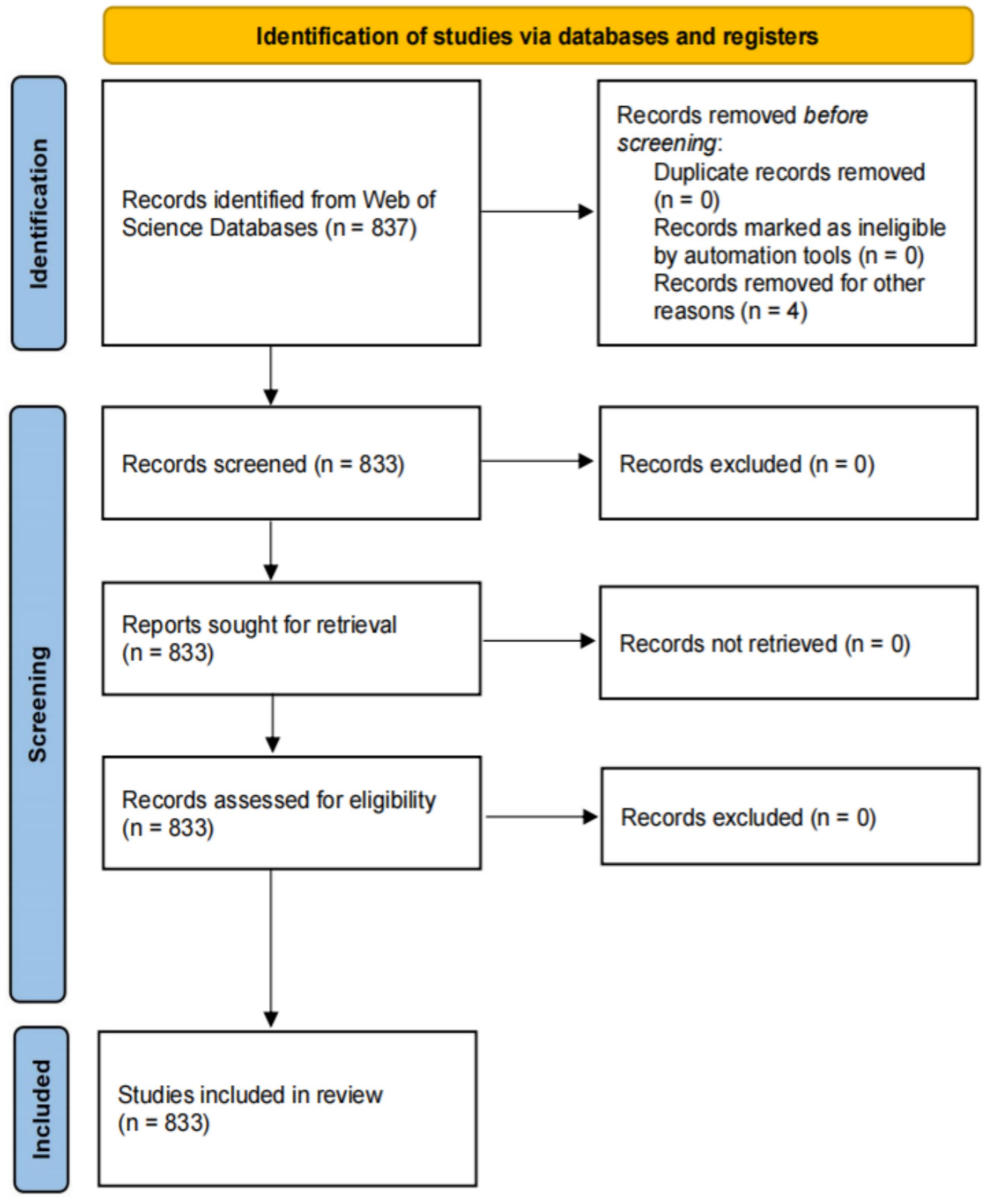
Flow chart of the study selection process.

There were no restrictions on the species or organisms studied, and duplicates were removed to ensure the dataset’s uniqueness. Ethical approval was not required, as the articles contained no personal patient information. The workflow diagram for this process is presented in Figure 1.

### 2.2 Literature selection

Two reviewers independently evaluated the articles. The initial screening involved reviewing titles and abstracts, followed by the application of predetermined inclusion and exclusion criteria. In cases of disagreement, a third assessor conducted a full review of the article and made the final decision regarding inclusion. The third assessor retained the authority to overrule any prior decisions. ① The inclusion criteria were: studies published in English between January 1, 1995 and January 10, 2024; original research articles and review articles focusing on the relationship between RA and oral bacteria/microbiome; studies investigating oral microbiota composition in RA patients; research on mechanisms linking periodontitis-associated bacteria to RA pathogenesis; clinical trials examining oral microbiome interventions in RA; and studies on specific oral pathogens (*P. gingivalis*, *A. actinomycetemcomitans*, *Prevotella*, etc.) in RA context. ② The exclusion criteria were: conference abstracts, letters to editor, case reports, book chapters, and editorial materials; studies focusing only on RA without oral microbiome aspects; studies focusing only on oral bacteria without RA connection; articles discussing only treatment outcomes without microbiome consideration; duplicate publications; articles without accessible full text; and articles with incomplete methodological description.

### 2.3 Analysis tool and parameter setting

The retrieved documents were exported in “plain text” format and renamed as “download_*.” CiteSpace 6.2.R4 and Excel 2016 were employed to analyze the co-occurrence network of authors, countries, institutions, and keywords within the included literature. The parameter settings for the CiteSpace software were as follows: time slicing ranged from 1995 to 2024; year per slice was set to 1 year, and the top *N* per slice was configured to 50. Pruning utilizes both the pathfinder method and pruned sliced networks. For the author, country, and institution analyses, no cutting method was selected, and the remaining parameters were maintained at their default values.

## 3 Results

### 3.1 Characteristics and trends in the volume of articles published

**Figure 2:**
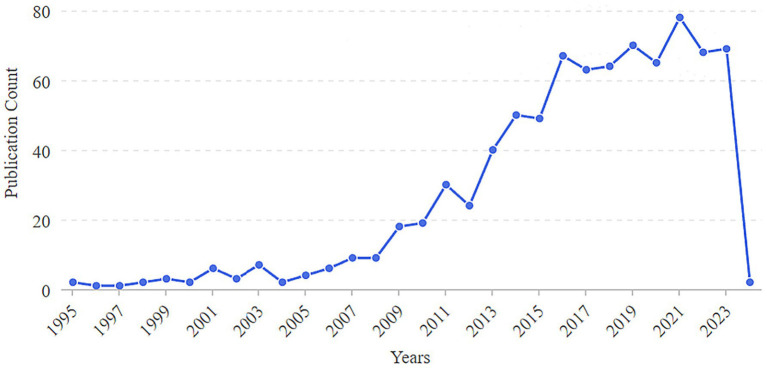
Annual number of publications on the correlation between rheumatoid arthritis and oral microbiome.

A total of 833 studies were included in this study. The period from 1995 to 2008 represented the initial stage, during which the number of published articles was less than 10. The period from 2009 to 2024 marks the development stage, which is characterized by a significant increase in published articles. Notably, in 2009, the number of published articles doubled compared to 2008. The year 2021 saw a publication peak, with 78 articles each. Although the number of published articles exhibited fluctuations throughout the period from 1995 to 2024, the overall trend indicates a steady increase in the annual volume of published articles (Figure 2).

### 3.2 Analysis of highly cited articles

Highly cited documents represent, to a certain extent, significant foundational text in this research field (Tian et al., 2017). Among the literature concerning the relationship between RA and the oral microbiome from 1995 to 2023, the most frequently cited works were published between 2013 and 2017 (Table 1). These documents illustrate the intricate interactions between RA and the oral microbiome, encompassing periodontitis, microbiome imbalances, and immune responses, which play a trend-setting role in the field. The article “Periodontitis: from microbial immune subversion to systemic inflammation” by Hajishengallis G. (2015) is the most cited, with 1,538 citations, underscoring its central importance in the investigation of the relationship between periodontitis and systemic inflammation. This study emphasizes the impact of periodontitis on systemic health, including RA, elucidates its mechanisms of action, and discusses the potential role of *P. gingivalis* in the production of autoantibodies associated with RA. Furthermore, this study posited that host-modulating therapies may be more effective than antibacterial approaches, garnering significant attention in the field (Sun et al., 2023).

**Table 1:** Top 10 cited documents.

Rank	First author	Year	Title	Total number of citations
1	Hajishengallis G.	2015	Periodontitis: from microbial immune subversion to systemic inflammation	1,538
2	Scher J. U.	2013	Expansion of intestinal *Prevotella copri* correlates with enhanced susceptibility to arthritis	1,272
3	Zhang X.	2015	The oral and gut microbiomes are perturbed in rheumatoid arthritis and partly normalized after treatment	1,042
4	Khandpur R.	2013	Nets are a source of citrullinated autoantigens and stimulate inflammatory responses in rheumatoid arthritis	888
5	Larsen J. M.	2017	The immune response to *Prevotella* bacteria in chronic inflammatory disease	649
6	Han Y. W.	2015	*Fusobacterium nucleatum*: a commensal-turned pathogen	452
7	Maeda Y.	2016	Dysbiosis contributes to arthritis development via activation of autoreactive t cells in the intestine	394
8	Konig M. F.	2016	*Aggregatibacter actinomycetemcomitans*-induced hypercitrullination links periodontal infection to autoimmunity in rheumatoid arthritis	346
9	Lee K. H.	2017	Neutrophil extracellular traps (nets) in autoimmune diseases: a comprehensive review	344
10	Han Y. W.	2013	Mobile microbiome: oral bacteria in extra-oral infections and inflammation	327

### 3.3 High-impact author collaboration features

**Figure 3:**
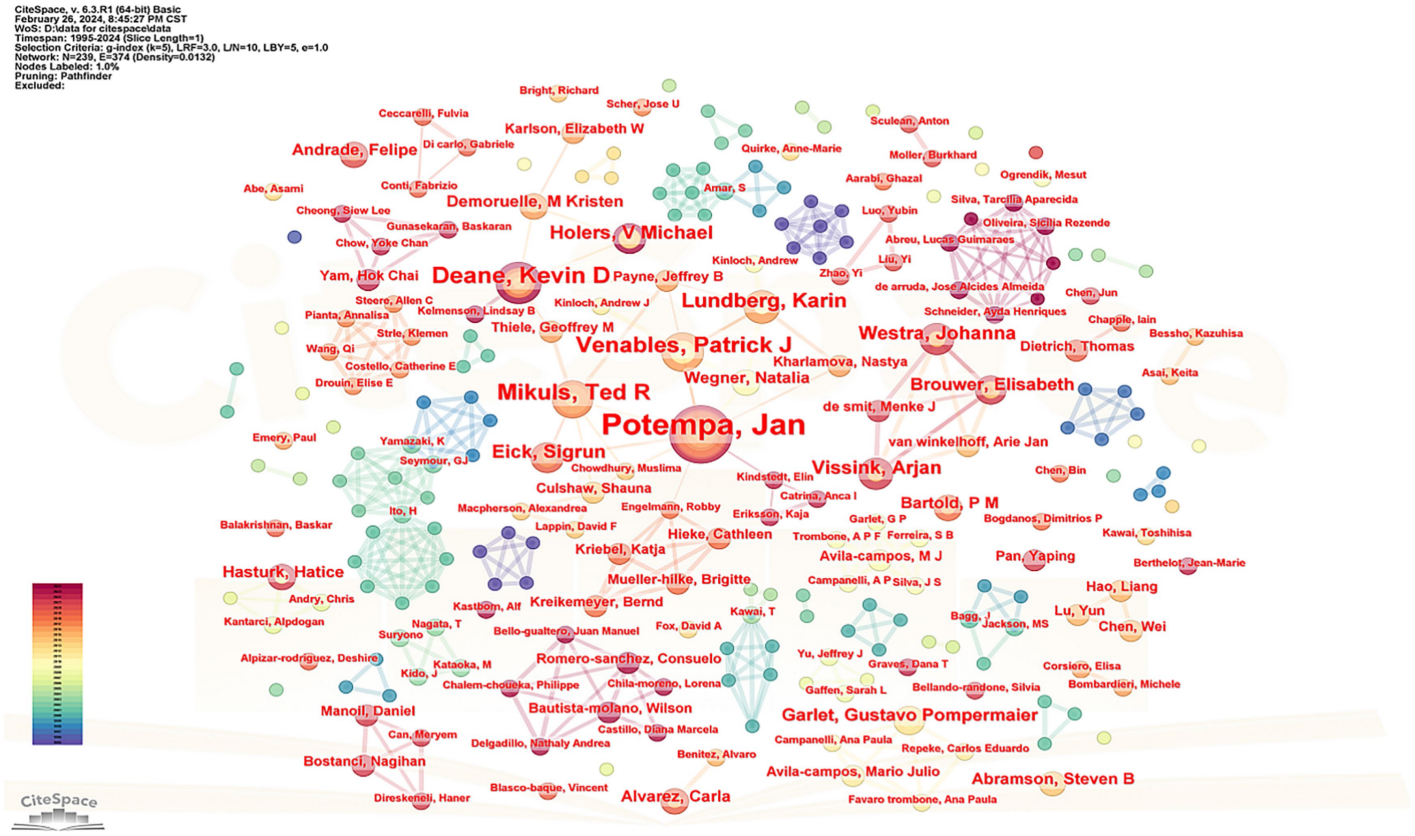
Map of author collaboration network analysis.

Figure 3 presents a cluster map of the author cooperation networks. The size of each node corresponds to the number of articles published by the authors, whereas the connecting lines between nodes represent cooperative relationships. Thicker lines indicate closer connections between the authors. The network comprised 289 nodes and 494 connections, illustrating the collaborative nature of the author teams.

Supplementary Table 1 provides information on the top five scholars, based on the number of articles published. Among these, Professor Jan Potempa from the United States published the most articles (31), while Professor Patrick J. Venables from Oxford University in the United Kingdom received the highest number of citations (2222), with an average citation count of 148.13 times. According to Price’s law, the minimum number of articles published by core authors is calculated (Hajishengallis, 2015) using the formula $M\approx 0.749\times \sqrt{n_{\max}}$ , where M represents the minimum number of articles published by core authors, and *n_max_* denotes the number of documents published by the author with the largest output. With *n*_max_ calculated as 31, M ≈ 4.17, indicating that authors with more than five papers are considered core authors. A total of 102 core authors published 313 articles, accounting for 37.6% of the total, which is less than 50%, suggesting that a cohesive core author group is yet to be established.

### 3.4 Country cooperation characteristics

**Figure 4:**
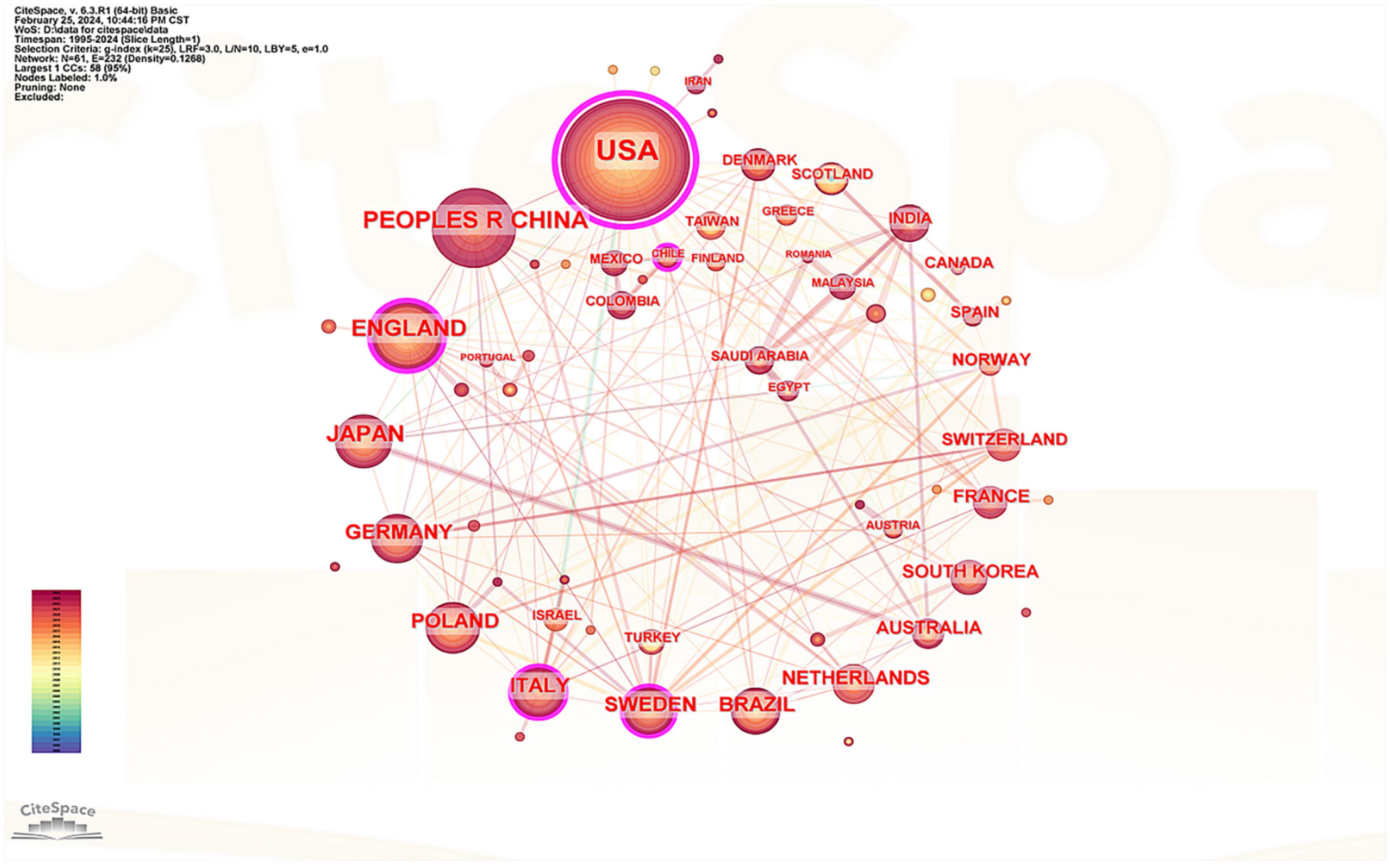
Knowledge map of countries cooperation network.

The cooperation network of countries that have published literature in this field was analyzed, encompassing 61 nations in the visualization. The top five countries identified were the United States, China, the United Kingdom, Japan, and Germany (see Supplementary Table 2). Notably, 80% of these leading countries are developed nations, which underscores the significant disparity in research output between advanced and developing countries. Figure 4 illustrates that the United States leads with 265 published articles, accounting for 31.81% of the total, with 22,171 citations, an average citation count of 83.66, and a centrality index of 0.89, all of which denote its substantial influence in this discipline. While China ranks second in terms of published articles, its average citation count is considerably lower than that of other leading countries, indicating a need for further enhancement in the research depth concerning the correlation between RA and the oral microbiome. Based on the centrality index, the top five countries were the United States (0.89), Britain (0.24), Italy (0.18), Sweden (0.1), and Germany (0.06). Connections between countries represent cooperative relationships, with thicker lines indicating closer ties. Figure 4 reveals strong cooperation between Japan and Australia as well as robust collaborative links between European nations, Europe, and Asia.

### 3.5 Characteristics of research institution cooperation

**Figure 5:**
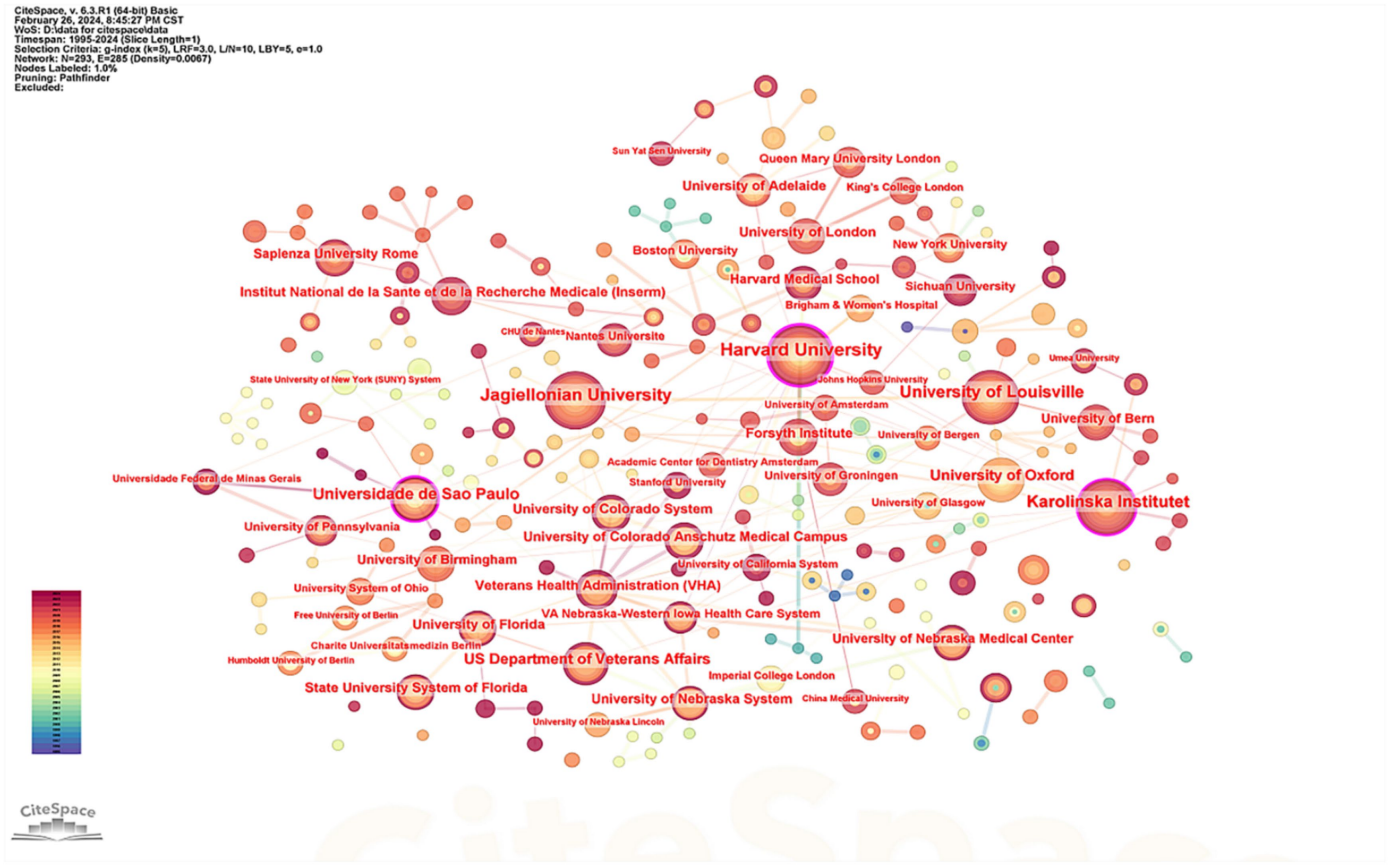
Knowledge map of institutional cooperation network.

The top five institutions identified in this study were Harvard University ( n = 36), Jagiellonian University ( n = 36), University of Louisville ( n = 32), Karolinska Institute ( n = 29), and the Universidade de São Paulo ( n = 25). Analysis of the number of published articles alongside citation counts indicates that Harvard University holds a leading position in this field. Supplementary Table 3 presents detailed information about the top five institutions based on the number of articles published, and Figure 5 visually represents these data.

### 3.6 Analysis of keywords

#### 3.6.1 Hotspot analysis of keywords in literature

**Figure 6:**
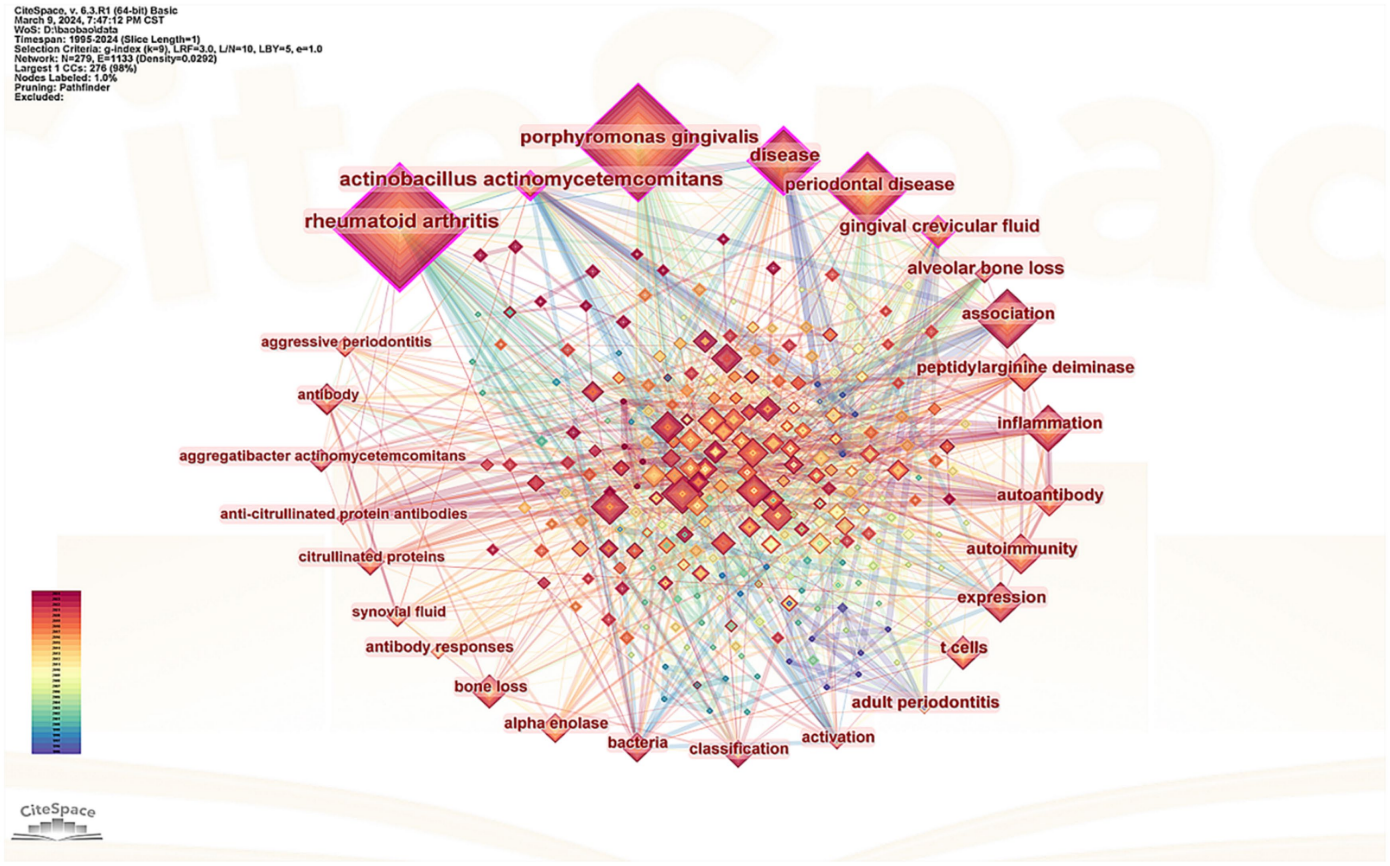
Map of keyword co-occurrence.

Keywords serve as concise summaries of an article’s content providing an intuitive understanding of key themes and research directions within a given field. The size of a node represents the frequency and significance of the keyword indicating its status as a hot topic in the current research. Additionally the thickness of connections between keywords reflects their interrelatedness. In this study 279 nodes and 1,133 connections. After excluding the keywords “rheumatoid arthritis” and “arthritis,” the top 10 keywords identified were *P. gingivalis* PD inflammation expression peptidylarginine deiminase (PAD) gut microbiota gingival crevicular fluid (GCF) antibody *Actinobacillus actinomycetemcomitans* (*A. actinomycetemcomitans*) and T cells (Figure 6).

#### 3.6.2 Analysis of keyword clusters

Keyword co-occurrence analysis was utilized to perform a cluster analysis of the keywords. The term extracted from the “keyword” served as the “cluster name,” visualized using the “log-likelihood rate” algorithm. The modularity value ( Q ) represents the significance of the clustering network while the silhouette value ( S ) evaluates the reliability of the clustering results. Typically a Q value above 0.3 indicates a meaningful clustering structure and an S value over 0.7 signifies a reliable cluster (Wildgaard et al., 2014).

**Figure 7:**
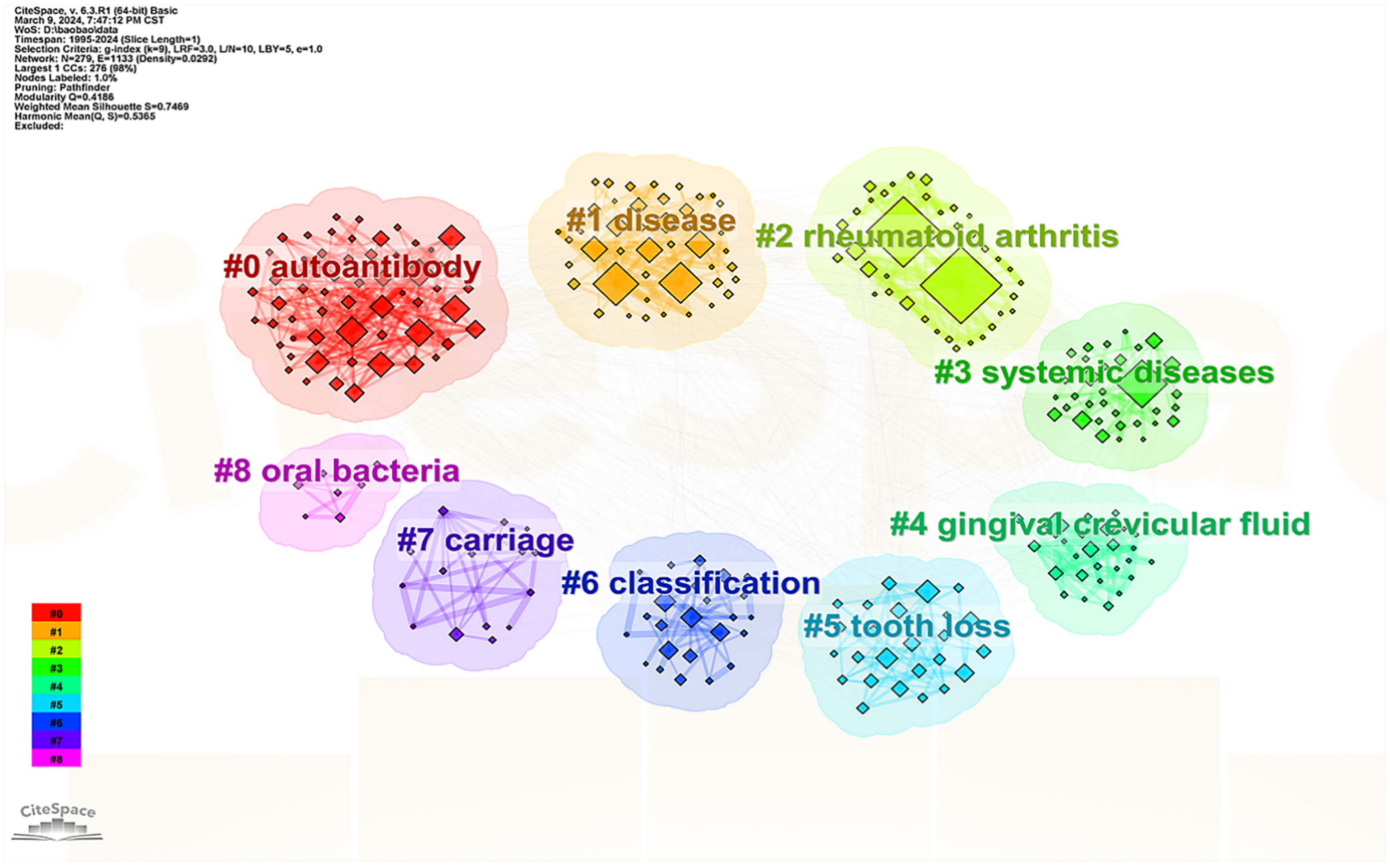
Map of keyword clustering.

In this study, the Q value for the cluster was 0.4186 (≥ 0.3) and the S value was 0.7469 (>0.7), confirming both the significance and credibility of the cluster structure. The identified clusters included #0 autoantibody, #1 disease, #2 RA, #3 systemic disease, #4 GCF, #5 tooth defect, #6 typing, #7 carrier, and #8 oral bacteria (Figure 7).

**Figure 8:**
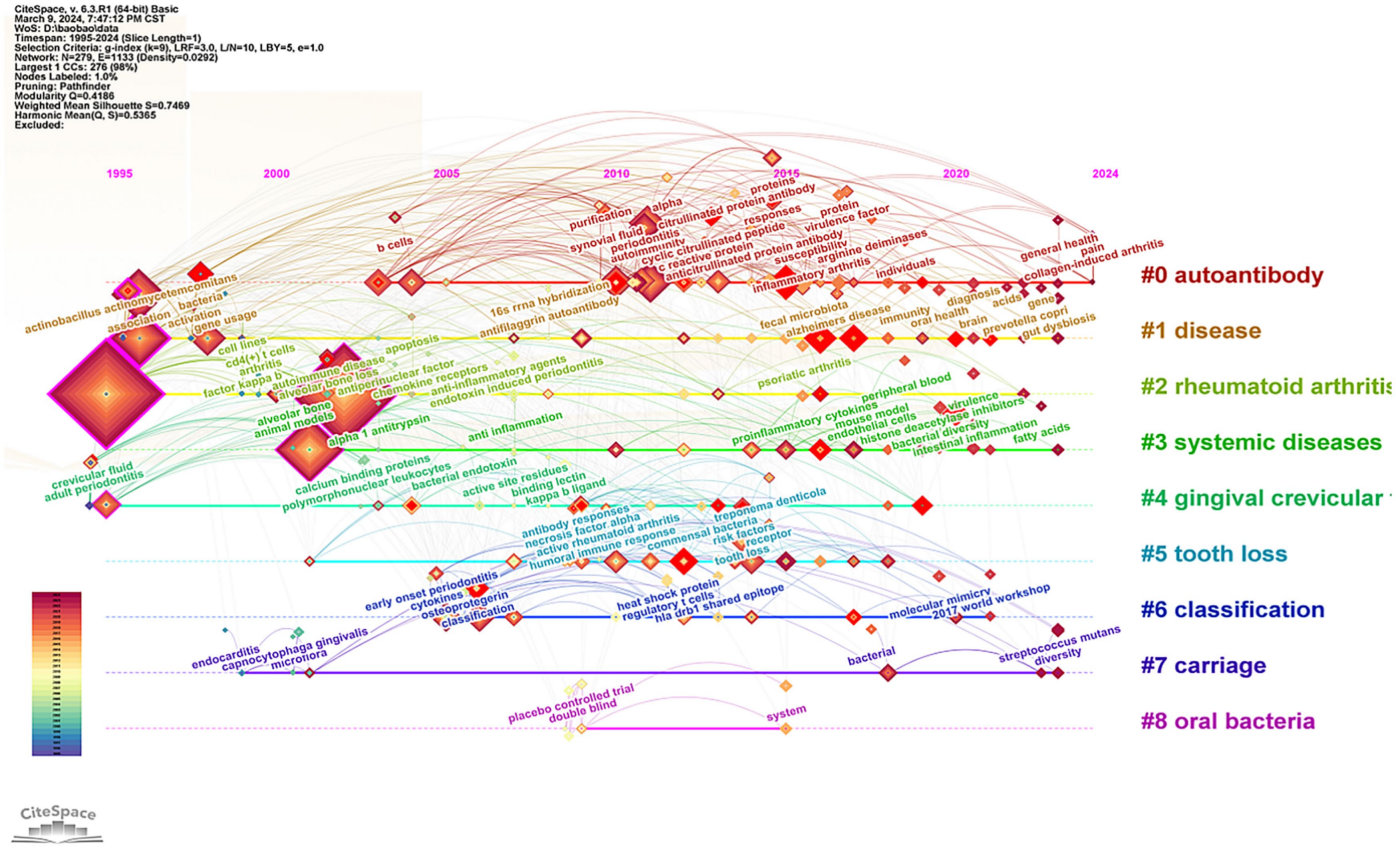
Visualization of timeline viewer of keyword clusters.

The keywords were distributed and displayed in a timeline diagram (Figure 8). This timeline graph illustrates the relationships between clusters and the historical spans of keywords within these clusters. Nodes belonging to the same cluster were arranged in chronological order along a horizontal line, with the time axis positioned above the view, extending sequentially from left to right. Clusters #0, #1, #3, and #7 are trending towards 2024, indicating that the influence of certain oral bacteria, such as *Streptococcus mutans*, on the progression of RA has emerged as a significant research trend in recent years. Conversely, the timelines of clusters #4, #5, and #8, spanning approximately 1996 to 2019, reflect a gradual decline in research related to gingival crevicular fluid and tooth defects in the context of RA. Additionally, the number of double-blind randomized controlled studies examining the relationship between RA and oral bacteria has been declining.

#### 3.6.3 Analysis of keyword trends emerging

**Figure 9:**
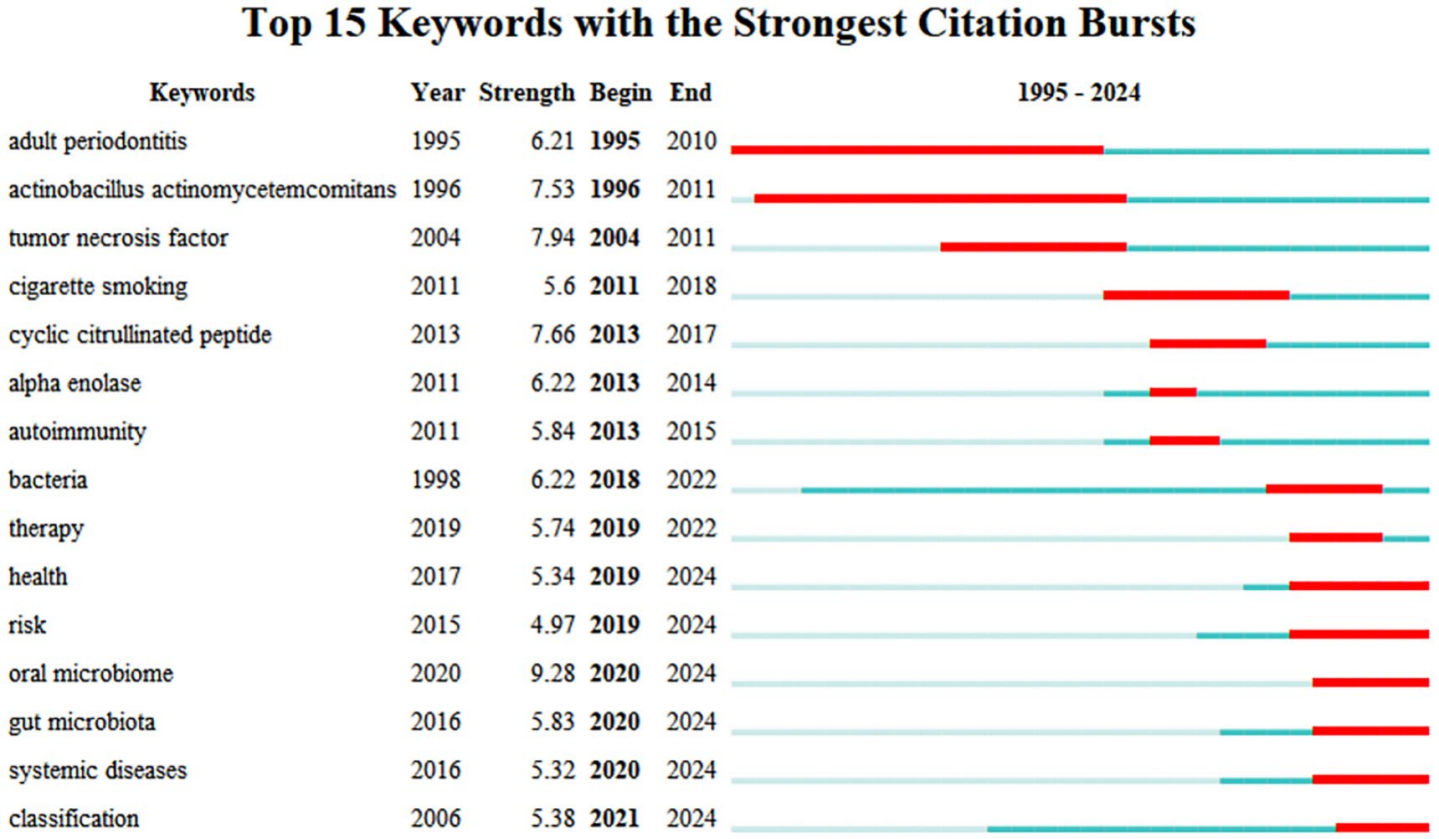
Top 15 keywords with the strongest citation bursts.

Keyword emergence refers to the phenomenon of keywords appearing frequently over a specific period reflecting the research hotspots in a particular field at different times and potentially predicting future research trends within that domain. The literature concerning the relationship between RA and the oral microbiome can be categorized into three main sections based on their chronological order. The first section (1995–2011) concentrates on the impact of oral health particularly periodontitis on RA emphasizing key bacteria within the oral microbiome and their relationship with inflammatory factors such as TNF. These studies aimed to elucidate the complex interactions between oral health and RA potentially providing intervention strategies to enhance the overall health of patients with RA. The second section (2011–2018) underscores the influence of smoking on the autoimmune system of patients with RA. This section indicates that smoking may affect both the immune system and oral microbiome of RA patients through various mechanisms with these factors collectively influencing disease progression and management. Research conducted in the past 5 years has increasingly focused on several aspects: health risk assessment the role of oral and intestinal microbiomes the connection between systemic diseases and the improved understanding and management of the interactions among these complex factors through classification methods. These trends suggest that researchers are actively investigating the impact of microbiota on RA and its implications in health risk management (Figure 9).

## 4 Discussion

### 4.1 Basic information

The annual number of publications in the literature serves as a crucial indicator for assessing the progress of academic research. Since 1995, scholars have explored the correlation between RA and oral pathogens. By 2009, the number of related publications had significantly increased, indicating a steady upward trend in research concerning the relationship between RA and the oral microbiome. This trend suggests that as research deepens and scholarly interest in this area grows, the volume of relevant literature correspondingly increases. Although there are peaks in publication activity in 2021, the number of publications has experienced a slight decline over the past 2 years, which may reflect the gradual maturation of research topics and the emergence of new research questions. Notably, research on the relationship between RA and the oral microbiome has garnered increasing attention in recent years, particularly regarding how the oral microbiome influences the mechanisms and treatment of RA. However, this research is predominantly concentrated in Europe and the United States, with most collaborations occurring internally between authors and institutions. There is a notable absence of core authors and institutions, and cross-team, cross-institution, and cross-regional collaboration remains relatively insufficient. This situation underscores the need for enhanced international cooperation and exchanges to collaboratively advance research progress in this field.

### 4.2 Research hotspot on the relationship between RA and oral microbiome

Keyword co-occurrence analysis was used to identify research hotspots in this field. The main keywords identified included *P. gingivalis* PD inflammation expression PAD gut microbiota GCF antibody *A. actinomycetemcomitans* and T cells. Numerous clinical epidemiological and serological studies have highlighted a connection between PD and the severity and progression of RA. While the etiologies of these diseases differ significantly strong links have been observed particularly regarding shared genetic and environmental risk factors. Among these factors *P. gingivalis* has emerged as a key contributor to PD pathogenicity (Yuan and Lai, 2023).

One of the critical bacterial factors linking *P. gingivalis* to RA is PAD, an enzyme whose structure and function have been extensively studied (Krutyhołowa et al., 2022). PAD catalyzes the transformation of arginine residues in proteins into citrulline (Bereta et al., 2019). This process, known as citrullination, can significantly impact protein structure and function. Dysregulated protein citrullination caused by microbial enzymes may disrupt normal host cell signaling, immune responses, and other homeostatic processes. In 2010, Professor Natalia Wegner and colleagues shed light on the autoimmune response to specific citrullinated proteins (e.g., fibrinogen, vimentin, type II collagen, and α-enolase), demonstrating that *P. gingivalis* uniquely produces PAD-citrullinated proteins (Zhu et al., 2022). However, the specific pathways and regulatory mechanisms by which *P. gingivalis* and its PAD contribute to RA pathogenesis remain unclear, necessitating further investigation in future studies.

The second most prevalent oral bacterium associated with RA pathogenesis is *A. actinomycetemcomitans*. These bacteria disrupt the balance of PAD activity in host neutrophils by secreting toxins, such as toxin P or leukotoxin A (LtxA), resulting in excessive protein citrullination and the formation of autoantigens within neutrophils. This imbalance plays a pivotal role in the loss of immune tolerance and the subsequent production of autoantibodies in RA (Wegner et al., 2010).

However, the exact mechanism by which LtxA triggers excessive citrullination in neutrophils remains unclear, as the specific neutrophil proteins regulating LtxA activity have yet to be identified. Further molecular investigations are needed to uncover the key proteins involved in this process. Given the role of *A. actinomycetemcomitans* in promoting citrullination, it is hypothesized that the PAD enzyme is a crucial factor likely upregulated during this process. While some studies have illuminated the role of PAD4 in RA-related citrullination, the potential impact of excessive PAD2 activation on cellular citrullination warrants deeper exploration (Konig et al., 2016; Looh et al., 2022).

While *P. gingivalis* and *A. actinomycetemcomitans* remain central to the discussion, emerging evidence suggests that other oral bacteria also play significant roles in RA pathogenesis. For example, *Prevotella intermedia* has been associated with elevated levels of anti-citrullinated protein antibodies (ACPAs) in RA patients, particularly in those with periodontitis. Similarly, *Fusobacterium nucleatum*, a commensal-turned-pathogen, has been shown to activate inflammatory pathways and exacerbate systemic immune responses. Recent studies have also identified *Tannerella forsythia* and *Megasphaera* spp. as potential contributors to RA severity. These findings underscore the complexity of the oral microbiome’s involvement in RA and highlight the need for further research into the virulence mechanisms of these organisms. Understanding the collective impact of multiple oral pathogens may provide new insights into disease progression and therapeutic strategies (Konig and Andrade, 2016; Kriebel et al., 2018).

Interestingly, one study revealed that only ACPA-positive RA is associated with *A. actinomycetemcomitans*-induced hypercitrullinated cells, as evidenced by elevated serum ACPA levels. In contrast, the connection between ACPA-negative RA and *A. actinomycetemcomitans* remains unclear, raising the possibility that this bacterium may not play a role in these cases, or that other pathogens could be involved (Lopez-Oliva et al., 2018). The inconsistent findings regarding the significance of *A. actinomycetemcomitans* in RA may stem from the lack of distinct group analysis in some studies. To clarify these associations, further research is needed to equally divide RA patients into ACPA-positive and ACPA-negative groups, enabling a comparative investigation of oral bacteria that might influence these two subsets of RA.

### 4.3 Global trend on the relationship between RA and oral microbiome

Based on the analysis of keyword emergence, early research primarily focused on the impact of oral health on RA. In recent years, the focus has gradually shifted towards smoking, health risk assessment, and the roles of the oral and intestinal microbiome. Keywords that have emerged prominently in recent studies include health, risk, oral microbiome, gut microbiota, systemic diseases, classification, and treatment. This shift indicates that researchers are beginning to pay greater attention to complex multifactor interactions and explore new intervention strategies.

#### 4.3.1 Oral microbiome

Research on the relationship between oral microorganisms and RA has been explored across various levels. A recent study utilized metagenomic association analysis of the oral microbiome to develop a diagnostic classification model that distinguished healthy individuals from RA patients with nearly 100% accuracy (van der Woude et al., 2010). This highlights a strong link between oral microbiome and the occurrence, progression, and prognosis of RA.

In patients with RA, the oral microbiome exhibits significant dysbiosis, and managing RA may help restore microbial equilibrium (van der Woude et al., 2010). Oral microorganisms are thought to contribute to RA onset and progression by inducing specific ACPA responses, activating toll-like receptor signaling pathways, and promoting T helper cell differentiation (Konig and Andrade, 2016; Kriebel et al., 2018; Edlund et al., 2017). Research has also shown that extensive citrullination occurs within the oral cavity, with citrullinated epitopes of oral microbes being targeted by highly somatically hypermutated ACPAs produced by RA plasma blasts, potentially driving RA pathogenesis (Brentano et al., 2005).

Previous studies have indicated that therapies targeting oral microbiome infections can enhance the condition of patients with RA. A proper diet, use of probiotics, and stringent oral hygiene practices may slow the progression of RA and mitigate the severity of the disease (Brewer et al., 2023; Nelson et al., 2020). However, research on the oral microbiome remains in its early stages, and it is currently uncertain whether *P. gingivali* and other bacteria associated with periodontal disease influence RA pathogenesis. While many studies have focused on uncovering microbial diversity and its virulence mechanisms, few have addressed the effects of community function, host genetic background, lifestyle, and biological functional events on the oral microbiome. The absence of these data significantly hampers a comprehensive understanding of the oral microbial communities. Well-designed randomized controlled trials and innovative research protocols are urgently needed to accurately assess the impact of antimicrobial therapy on outcomes and responses to RA treatment. Moreover, resolving outstanding clinical questions should be predicated on a thorough understanding of the mechanisms underlying epidemiological associations. Identifying oral microorganisms and their mechanisms related to RA is essential for advancing future research on the treatment, diagnosis, and prevention of RA.

#### 4.3.2 Gut microbiota

A stable gut microbiota is essential for various physiological functions, including fostering immune system development and homeostasis (Koziel and Potempa, 2022). Dysbiosis of gut microbiota has been implicated in autoimmune diseases such as RA. Among the gut microbes, Prevotella has garnered significant attention in RA research. Although *P. gingivalis* has been detected in the feces of RA patients, its abundance is relatively low. Interestingly, studies have found a positive correlation between the abundance of *P. gingivalis* and the genus Prevotella (Sato et al., 2017), suggesting a potential synergistic interaction between these microbes in the gut microbiota of RA patients. Importantly, one study strongly supports the hypothesis that Prevotella may act as a causative pathogen in the development of RA (Kishikawa et al., 2022).

Some researchers have explored the pathways and factors through which the oral microbiome influences the gut microbiota and how their interaction may impact systemic diseases. Studies have shown that oral bacteria such as *P. gingivalis* can translocate from the oral cavity to the gastrointestinal tract through aspiration or swallowing, where they may colonize and alter the composition and function of the gut microbiota (Tan et al., 2023). This influence primarily manifests as changes in microbial diversity, including a reduction in beneficial commensals like Bifidobacterium and an increase in pro-inflammatory taxa such as *Prevotella*. Moreover, these oral-derived bacteria may modulate host immune responses through metabolic activities, such as the production of short-chain fatty acids or lipopolysaccharides, thereby affecting intestinal permeability and systemic inflammation.

The concept of the oral-gut axis in systemic diseases has recently emerged, proposing that *P. gingivalis* could connect RA and PD by affecting the intestinal immune system and altering gut microbiota composition (Pianta et al., 2017). This novel etiological mechanism along the oral-gut-systemic axis offers a plausible explanation for the link between periodontal disease and RA, expanding beyond traditional disease models.

Given these insights, this study uniquely positions itself as a foundation for future research by highlighting the interconnected roles of oral and gut microbiota in RA pathogenesis. While Namrata Dagli focused on the impact of RA on oral health, our findings extend beyond this scope by emphasizing the mechanistic pathways through which microbial communities influence systemic diseases. Future research should prioritize developing targeted interventions, such as probiotics or vaccines, to modulate these microbial communities and improve RA patient outcomes. This broader perspective underscores the novelty and significance of our study in advancing the understanding of RA and its relationship with the oral microbiome. These strategies might include the use of probiotics to reshape the microbial community, or the development of vaccines or therapeutics aimed at specific microorganisms.

### 4.4 Limitations of the study

Although we strictly adhered to the bibliometric analysis methods and research strategies, the current study has certain limitations. Relying exclusively on the Web of Science database to search for English-language articles published to date may lead to an incomplete literature search and introduce biases related to language and publication. Moreover, due to the nature of bibliometric analyses, this study primarily focuses on quantitative trends rather than in-depth qualitative interpretation of individual studies. However, one of the key strengths of this study lies in its application of CiteSpace software to visualize knowledge structures and identify emerging trends. Compared to traditional narrative reviews, the use of bibliometric tools enables a more systematic and objective analysis of global research patterns. This methodology is particularly applicable to other highly relevant topics in microbiology and current autoimmune diseases, where large-scale data synthesis and trend forecasting are critical. For instance, similar approaches could be extended to explore the microbial involvement in systemic lupus erythematosus, inflammatory bowel disease, or even the gut-lung axis in respiratory autoimmunity. By highlighting these broader applicability domains, our study not only contributes to RA and oral microbiome research but also provides a replicable framework for investigating complex host-microbe interactions in other autoimmune conditions. While there may be some overlap with prior reviews, such as the one by Namrata Dagli, this study offers a distinct contribution by employing advanced bibliometric tools to systematically analyze research trends, collaborative networks, and emerging topics in the relationship between RA and the oral microbiome. Our findings not only complement existing literature but also provide a roadmap for future research by identifying gaps and opportunities in the field. This approach ensures that our study serves as a valuable resource for scholars seeking to advance the understanding of RA and its complex interactions with microbial communities.

## 5 Conclusion

This study employed bibliometric methods to analyze the literature on the relationship between the oral microbiome and RA. We assessed literature from various years, countries, institutions, and authors, tracking the progress of this research field since 1995 while also making predictions about potential future research directions. The findings indicated a consistent annual increase in the number of publications within this domain. Notably, the United States, Harvard University, and Jan Potempa have had a significant influence on this area. The mechanisms by which oral and intestinal microorganisms affect RA, along with intervention strategies targeting these microbial communities, represent the frontiers and focal points of the current research.

In addition to analyzing the research trends and hotspots related to RA and the oral microbiome, this study utilized CiteSpace software, a powerful tool for visualizing scientific landscapes. CiteSpace enabled us to systematically map co-authorship networks, keyword clustering, and emerging trends over time. By generating knowledge graphs based on bibliographic data, it facilitated the identification of key contributors, major research themes, and evolving topics. This visualized approach not only deepened our understanding of the intellectual structure of the field but also provided scholars with a clear roadmap for future research directions. Therefore, the use of CiteSpace was instrumental in achieving the objectives of this study and highlighted the importance of integrating bibliometric tools into systematic reviews of complex biomedical topics.

## Data Availability

The original contributions presented in the study are included in the article/Supplementary material, further inquiries can be directed to the corresponding author.

## Footnotes

^1^ https://webofknowledge.com
