# Supplementary material for: Bibliometric and visualized analysis of the relationship between rheumatoid arthritis and periodontitis-related bacteria using CiteSpace software
Source: Front Microbiol. 2025 Jul 16;16:1589331. doi: 10.3389/fmicb.2025.1589331 (PMC12307397; doi:10.3389/fmicb.2025.1589331)
Supplement: Supplementary file 1 [file Table_1.docx]

Supplemental Table 1. Top five published authors.

| **Rank** | **Author** | **Number of articles issued** | **Frequency of citations** | **Average of citations** |
| --- | --- | --- | --- | --- |
|  |  |  |  |  |
| 1 | Potempa J | 31 | 2165 | 69.84 |
| 2 | Mikuls TR | 18 | 1494 | 83 |
| 3 | Venables PJ | 15 | 2222 | 148.13 |
| 4 | Lundberg K | 14 | 1733 | 123.79 |
| 5 | Deane KD | 13 | 1287 | 99 |

Supplemental Table 2. Top five countries by number of publications.

| **Rank** | **Country** | **Publication** | **Frequency of citations** | **Average of citations** | **Centrality** |
| --- | --- | --- | --- | --- | --- |
| 1 | United States | 265 | 22171 | 83.66 | 0.89 |
| 2 | China | 105 | 4161 | 39.63 | 0.04 |
| 3 | United Kingdom | 87 | 7087 | 81.46 | 0.24 |
| 4 | Japan | 78 | 2843 | 36.45 | 0.02 |
| 5 | Germany | 53 | 2204 | 41.58 | 0.06 |

Supplemental Table 3. Top five institutions with the most publications.

| **Rank** | **Institution** | **Publication** | **Frequency of citations** | **Average of citations** |
| --- | --- | --- | --- | --- |
| 1 | Harvard University | 36 | 3841 | 106.69 |
| 2 | Jagiellonian University | 36 | 2261 | 62.81 |
| 3 | University of Louisville | 32 | 2312 | 72.25 |
| 4 | Karolinska Institutet | 29 | 2093 | 72.17 |
| 5 | Universidade of São Paulo | 25 | 892 | 35.68 |
